# Spatial patterns, temporal trends, and age-period-cohort effects of colon cancer incidence in Kazakhstan, 2005–2024

**DOI:** 10.3389/fonc.2026.1732883

**Published:** 2026-05-19

**Authors:** Gulnur Igissinova, Nurbek Igissin, Askhat Axarin, Aram Halimi, Zhansaya Telmanova, Dulat Turebayev, Akzhunis Jexenova, Nurymzhan Murzagulov, Sergey Dyakov, Rustem Moldagali, Niiazbek Mamatov, Zarina Bilyalova

**Affiliations:** 1 Central Asian Institute for Medical Research, Astana, Kazakhstan; 2 Department of Oncology, Asfendiyarov Kazakh National Medical University, Almaty, Kazakhstan; 3 Research Institute of Life and Health Sciences, Higher School of Medicine, Kokshetau University named after Sh. Ualikhanov, Kokshetau, Kazakhstan; 4 Asian Pacific Organization for Cancer Prevention, Bishkek, Kyrgyzstan; 5 Department of Postgraduate Studies, Akhunbaev Kyrgyz State Medical Academy, Bishkek, Kyrgyzstan; 6 Professor G.V. Tsoi Scientific and Educational Center of Surgery, Astana Medical University, Astana, Kazakhstan; 7 Research Center for Social Determinants of Health, Research Institute for Metabolic and Obesity Disorders, Research Institute for Endocrine Sciences, Shahid Beheshti University of Medical Sciences, Tehran, Iran; 8 Department of Radiation Diagnostics, National Scientific Center of Traumatology and Orthopedics named after Academician Batpenov N.D. of the Ministry of Health of the Republic of Kazakhstan, Astana, Kazakhstan; 9 Kokshetau Higher Medical College, Kokshetau, Kazakhstan

**Keywords:** age-period-cohort analysis, colon cancer, incidence trends, joinpoint regression, Kazakhstan, spatial epidemiology

## Abstract

**Background:**

Colon cancer incidence is increasing globally, yet its spatial and temporal dynamics in Central Asia remain insufficiently studied. This study aimed to assess regional disparities and temporal trends of colon cancer incidence in Kazakhstan from 2005 to 2024 using geospatial and age-period-cohort analyses.

**Methods:**

A nationwide population-based study was conducted using data from the Unified Nationwide Electronic Health System of Kazakhstan. Crude incidence rates (CR) and age-standardized incidence rates (ASR) were calculated per 100,000 population using the WHO world standard population. Spatial patterns were assessed based on the administrative division of 2005, and clustering was evaluated using the Getis–Ord Gi* statistic. Temporal trends were analyzed using Joinpoint regression and Age-Period-Cohort analysis modeling, including estimation of net drift, local drift, and period and cohort rate ratios.

**Results:**

The national CR was 9.64 per 100,000, and the ASR was 9.44 per 100,000. A persistent north-south gradient was observed, with higher incidence in northern and central regions (Pavlodar, Kostanay, Karaganda, North Kazakhstan, Astana) and lower rates in southern regions. Spatial clustering analysis identified significant hotspots in northern regions and coldspots in the south. Age-Period-Cohort analysis demonstrated a strong age effect across all models. The net drift was +0.57% per year (95% CI 0.23–0.91; p=0.001), with the highest increases observed in older age groups (65–84 years). Period and cohort effects were also significant, indicating the influence of demographic aging, healthcare changes, and generational risk factors.

**Conclusion:**

Colon cancer incidence in Kazakhstan is characterized by pronounced spatial disparities and is primarily driven by population aging. The combined geospatial and Age-Period-Cohort analysis approach provides a comprehensive framework for understanding disease dynamics and supports the development of targeted cancer control strategies.

## Introduction

Colorectal cancer is one of the leading causes of cancer-related morbidity and mortality worldwide, ranking third in incidence and second in mortality globally (1). Colon cancer, as a major component of colorectal malignancies, demonstrates substantial geographic variability influenced by socioeconomic development, healthcare accessibility, and population risk profiles (2).

In 2020, there were over 1.9 million new colorectal cancer cases and 935,173 colorectal cancer-related deaths worldwide, with males accounting for 55.1% of all cases. The global colorectal cancer age-standardized incidence rate (ASR) was 19.5 per 100,000 (23.4 in males, 16.2 in females). Furthermore, the overall colorectal cancer age-standardized mortality rate (ASMR) was 9 per 100,000 (11 in males, 7.2 in females). Incidence rates were highest in Australia/New Zealand and European regions (40.6 per 100 000, males) and lowest in several African regions and Southern Asia (4.4 per 100 000, females). Similar patterns were observed for mortality rates, with the highest observed in Eastern Europe (20.2 per 100 000, males) and the lowest in Southern Asia (2.5 per 100 000, females). The burden of colorectal cancer is projected to increase to 3.2 million new cases and 1.6 million deaths by 2040 with most cases predicted to occur in high or very high human development index (HDI) countries (3, 4).

In Asia, colorectal cancer incidence has been rising rapidly, especially in East Asia and South-East Asia. In 2020, ASR of CRC in Japan was 38.2 per 100,000, while South Korea had a significantly lower rate of 26.9 per 100,000. These findings are mostly attributed to increasing rates of smoking, alcohol intake, and red meat diet in East Asia, which are established risk factors for gastrointestinal cancers (5).

In Kazakhstan, the overall age-standardized mortality rate of colorectal cancer have decreased, which shows a decline from 11.2 to 7.7 deaths per 100,000 population (6). Zhylkaidarova et al. (2021) examined trends in colorectal cancer (CRC) in Kazakhstan, emphasizing the effects of the nationwide screening program implemented in 2011. The study indicated a notable rise in colorectal cancer incidence, especially in the early stages (I and II). The incidence rates of stages I and II exhibited a two-fold increase (35-67.4%), while the incidence of stage IV decreased from 19.3% to 13.1%, and stage III declined from 45.7% to 19.5% from 2004 to 2018, respectively. The total incidence of colorectal cancer in Kazakhstan increased to 18.7 per 100,000 by 2018, exhibiting notable geographical disparities. Regions with high morbidity, such as Almaty and North Kazakhstan, experienced significant increases in detection rates after the initiation of screening, but localities like Zhambyl and Qyzylorda exhibited lower rates despite the adoption of the screening program (7).

This study aimed to characterize the spatial and temporal epidemiology of colon cancer incidence in Kazakhstan over the period 2005-2024, with particular emphasis on age- and sex-specific burden, regional structural shifts, geographic clustering, and the contributions of age, period, and cohort effects to the observed incidence patterns.

## Materials and methods

### Study design and setting

A nationwide retrospective population-based study was conducted to assess colon cancer incidence in Kazakhstan from 2005 to 2024. The analysis focused exclusively on colon cancer (ICD-10 code C18). Colon and rectal cancers were not pooled because they differ in anatomical subsite, age distribution, screening detectability, and epidemiological behavior. All analyses were performed for the total population and stratified by sex, age, and region.

### Data sources, case definition, and population denominators

Incident colon cancer cases were extracted from the Unified Nationwide Electronic Health System (UNEHS) of the Republic of Kazakhstan. The study included all newly registered primary cases of colon cancer recorded during 2005–2024 among permanent residents of Kazakhstan. Follow-up encounters were not treated as new incident cases. Regional assignment was based on the patient’s place of permanent residence rather than place of treatment.

Annual population denominators by age, sex, and region were obtained from the Bureau of National Statistics of the Republic of Kazakhstan. To ensure comparability over time, all spatial analyses were harmonized to the administrative-territorial division of Kazakhstan as of the beginning of the study period (2005), and later administrative changes were back-mapped to these baseline boundaries.

### Age stratification and incidence measures

Incidence was analyzed overall, by sex, by region, and by age. Age-specific rates were calculated in 5-year age groups. For descriptive, spatial, and trend analyses, age was additionally aggregated into two clinically and epidemiologically relevant categories:<50 years and ≥50 years, representing early-onset and later-onset disease, respectively.

Crude incidence rates (CR) were calculated per 100,000 population as:


$$CR=\frac{N}{P}\times 100,000$$

where *N* is the number of incident cases and *P* is the corresponding population.

Age-specific incidence rates were calculated as:


$$r_{i}=\frac{n_{i}}{p_{i}}\times 100,000$$

where *n_i_* and *p_i_* are the number of cases and population in the *i*-th age group

Age-standardized incidence rates (ASR) were estimated by the direct method using the WHO world standard population (8):


$$ASR=\frac{\sum \left(r_{i}\times w_{i}\right)}{\sum w_{i}}$$

where *r_i_* is the age-specific incidence rate and *w_i_* is the corresponding weight in the standard population.

All incidence indicators were expressed per 100,000 population. For descriptive summaries over the study period, total case counts, proportions, and mean annual crude and age-standardized rates were calculated for the total population and separately by sex, age group, and region.

### Regional structural analysis and crude-standardized gap

To examine regional contributions to the national case burden, the share of each region in the total number of incident colon cancer cases in Kazakhstan was calculated as:


$$\text{Regional share}=\frac{\text{cases in region}}{\text{cases in Kazakhstan}}\times 100$$

Structural changes between 2005 and 2024 were expressed in percentage points (p.p.) and were evaluated for the total population and separately for the<50- and ≥50-year age groups.

To assess the contribution of regional age structure to the observed burden, the crude–standardized gap was calculated as:


Δ=CR−ASR

Positive values indicated a greater contribution of older population structure to crude incidence, whereas negative values suggested a relatively younger regional age composition.

### Temporal trend analysis

Temporal trends in colon cancer incidence were assessed using Joinpoint Regression Program (National Cancer Institute, USA), version 5.4.0. Annual crude and age-standardized incidence rates were modeled on the log-linear scale. Annual percent change (APC) and average annual percent change (AAPC) with 95% confidence intervals were estimated.

Model selection was based on the weighted Bayesian Information Criterion, allowing from 0 to 3 joinpoints. Statistical significance was evaluated using the Monte Carlo permutation method with a two-sided significance level of 0.05 and up to 4,499 permutations. Standard errors were estimated assuming a Poisson distribution of case counts.

### Spatial cartography and cluster analysis

Descriptive spatial analysis was based on both crude and age-standardized regional incidence rates. Regional classification was based on the national mean (*x*) and standard deviation (σ) of the corresponding indicator, with categories defined as low ( <x−0.5σ), average ( x−0.5σ to x+0.5σ), and high ( >x+0.5σ) incidence. The cartographic visualization of thematic maps was performed using Datawrapper Maps (Datawrapper GmbH, Berlin, Germany).

• To assess spatial changes in relation to screening and health system phases, period-specific mean age-standardized incidence rates were calculated for five analytically defined intervals: Pre-screening baseline (2005–2010): Absence of organized population-based screening; serves as a historical control (7).
• Early implementation (2011–2013): Launch of nationwide screening for ages 50–70; short-term incidence rise due to case detection (7).
• Program scaling and stabilization (2014–2019): Expansion under Salamatty Kazakhstan (2011–2015) (11) and Densaulyk (2016-2019) programs (12), and preparation of the Comprehensive Cancer Control Plan 2018-2022 (13).
• Pandemic shift (2020-2021): COVID-19-related reduction in screening coverage; modeled as a separate binary segment in time-series analysis (14).
• Recovery and modernization (2022-2024): Restoration of screening, regional adaptations, and sustainability assessment following completion of the Comprehensive Plan 2018-2022 (13). These intervals were defined *a priori* to reflect major stages of organized colorectal cancer screening and healthcare system disruption.

Spatial clustering was evaluated using the Getis-Ord Gi* statistic in ArcMap 10.8.2 (Esri, Redlands, CA, USA) (9, 10). The analysis was applied to regional period-specific mean ASR values using an adjacency-based polygon neighborhood structure. Regions with positive *z*-scores and statistically significant *p*-values were classified as hotspots, whereas regions with negative *z*-scores were classified as coldspots. Confidence levels of 90%, 95%, and 99% were used to interpret the strength of clustering.

No spatial smoothing was applied, because the analysis was based on aggregated multi-year regional rates rather than unstable single-year local rates. Given the limited number of spatial units and the regional scale of analysis, hotspot and coldspot findings were interpreted as exploratory clustering patterns rather than fine-scale local clusters. No formal multiple-comparison correction was applied to Gi* statistics.

### Age-period-cohort analysis

Age-period-cohort analysis was performed using the National Cancer Institute Age-period-cohort analysis Web Tool. Separate models were fitted for the total population, men, and women. Age-period-cohort analysis models were constructed using consecutive 5-year age groups and 5-year calendar periods. To ensure model stability, the Age-period-cohort analysis was restricted to ages 30–34 through 80–84 years; younger ages were excluded because of sparse counts, and the open-ended oldest age group was not modeled. Calendar periods were defined as 2005-2009, 2010-2014, 2015-2019, and 2020-2024. The reference period was 2010-2014, and the reference birth cohort was 1955.

The following Age-period-cohort analysis parameters were estimated: net drift, local drift, longitudinal age curve, period rate ratios, and cohort rate ratios. Net drift represents the overall annual log-linear change in incidence across periods and cohorts, while local drift reflects age-specific annual changes. Period and cohort effects were expressed as rate ratios relative to the respective reference categories.

### Ethics approval

The research employed publicly accessible administrative data, hence it did not entail direct engagement with individuals. Ethical permission was secured from the Local Ethics Commission of the Central Asian Institute for Medical Research.

## Results

The analysis was structured to evaluate colon cancer incidence across demographic, spatial, and temporal dimensions, including age-specific patterns, regional distribution, spatial clustering, and age–period-cohort effects.

### Age and sex distribution of colon cancer cases

During 2005-2024, 31,293 new cases of colon cancer were recorded in Kazakhstan, of which 45.4% occurred in men and 54.6% in women. The age profile was strongly shifted toward older adults: 89.0% of all cases were diagnosed in individuals aged ≥50 years, while those aged<50 years represented only 11.0% of the total case load (**Table 1**).

**Table 1:** Age and sex-specific distribution of colon cancer cases in Kazakhstan, 2005–2024 (n, % of total).

Age group	Both sexes, n (%)	Male, n (%)	Female, n (%)
<5	3 (0.01)	1 (0.00)	2 (0.01)
5-9	2 (0.01)	2 (0.01)	0 (0.00)
10-14	5 (0.02)	3 (0.01)	2 (0.01)
15-19	43 (0.14)	24 (0.08)	19 (0.06)
20-24	77 (0.25)	44 (0.14)	33 (0.11)
25-29	101 (0.32)	55 (0.18)	46 (0.15)
30-34	337 (1.1)	165 (0.5)	172 (0.5)
35-39	499 (1.6)	257 (0.8)	242 (0.8)
40-44	894 (2.9)	443 (1.4)	451 (1.4)
45-49	1472 (4.7)	692 (2.2)	780 (2.5)
50-54	2537 (8.1)	1165 (3.7)	1372 (4.4)
55-59	3808 (12.2)	1825 (5.8)	1983 (6.3)
60-64	4856 (15.5)	2319 (7.4)	2537 (8.1)
65-69	5533 (17.7)	2527 (8.1)	3006 (9.6)
70-74	4785 (15.3)	2139 (6.8)	2646 (8.5)
75-79	3684 (11.8)	1552 (5.0)	2132 (6.8)
80-84	1959 (6.3)	747 (2.4)	1212 (3.9)
85+	698 (2.2)	258 (0.8)	440 (1.4)
Total	31293 (100.0)	14218 (45.4)	17075 (54.6)
<50	3433 (11.0)	1686 (5.4)	1747 (5.6)
≥50	27860 (89.0)	12532 (40.0)	15328 (49.0)

The burden increased markedly with age, beginning from the fourth decade of life and rising steeply after age 50. The largest proportion of cases was concentrated in the 60-69-year age range, with a peak in the 65-69-year group (17.7%), followed by the 60-64-year (15.5%) and 70-74-year (15.3%) groups. Thereafter, the proportion declined progressively in older age categories (**Table 1**).

Women constituted a slightly greater proportion of total cases overall and predominated in the older age groups, particularly after 65 years. Thus, the descriptive age-sex structure of colon cancer incidence in Kazakhstan indicates a predominantly late-onset disease pattern with a substantial concentration in the retirement-age population (**Table 1**).

### Age-specific crude and age-standardized incidence rates

Age-specific incidence rates of colon cancer in Kazakhstan showed a strong and progressive increase with age. In the total population, rates remained below 1 per 100,000 before age 30, rose to 1.2 at 30–34 years, 6.8 at 45–49 years, and increased sharply thereafter to 13.0, 22.8, and 38.1 per 100,000 in the 50-54, 55-59, and 60-64-year age groups, respectively.

The incidence peaked at 75–79 years (78.9 per 100,000) and declined slightly in the oldest age groups (**Table 2**).

**Table 2:** Age-specific, crude and age-standardized incidence rates of colon cancer in Kazakhstan, 2005– 2024 (per 100,000 population).

Age group	Incidence, mean ± SE* [95% CI**]		
	Both sexes	Male	Female
<5	<0.1	<0.1	<0.1
5-9	<0.1	<0.1	<0.1
10-14	<0.1	<0.1	<0.1
15-19	0.2 ± 0.04 [0.1; 0.2]	0.2 ± 0.1 [0.1; 0.3]	0.1 ± 0.1 [0.1; 0.2]
20-24	0.3 ± 0.05 [0.2; 0.4]	0.3 ± 0.06 [0.2; 0.4]	0.3 ± 0.1 [0.1; 0.4]
25-29	0.3 ± 0.04 [0.3; 0.4]	0.4 ± 0.07 [0.2; 0.5]	0.3 ± 0.04 [0.2; 0.4]
30-34	1.2 ± 0.1 [1.1; 1.4]	1.2 ± 0.1 [1.0; 1.4]	1.2 ± 0.1 [1.1; 1.4]
35-39	2.0 ± 0.1 [1.8; 2.2]	2.1 ± 0.1 [1.8; 2.4]	1.9 ± 0.1 [1.6; 2.2]
40-44	3.9 ± 0.2 [3.6; 4.3]	4.0 ± 0.2 [3.7; 4.4]	3.9 ± 0.2 [3.4; 4.3]
45-49	6.8 ± 0.2 [6.4; 7.2]	6.7 ± 0.3 [6.2; 7.3]	6.9 ± 0.3 [6.4; 7.4]
50-54	13.0 ± 0.3 [12.4; 13.6]	12.8 ± 0.4 [12.0; 13.6]	13.1 ± 0.4 [12.3; 14.0]
55-59	22.8 ± 0.6 [21.7; 23.8]	24.1 ± 0.7 [22.6; 25.5]	21.7 ± 0.6 [20.5; 22.9]
60-64	38.1 ± 0.9 [36.3; 39.8]	41.9 ± 1.3 [39.3; 44.5]	35.2 ± 0.9 [33.3; 37.0]
65-69	57.6 ± 2.3 [53.1; 62.1]	65.4 ± 3.2 [59.1; 71.7]	52.5 ± 2.0 [48.6; 56.4]
70-74	67.7 ± 1.5 [64.8; 70.6]	81.8 ± 2.1 [77.6; 86.0]	59.4 ± 1.4 [56.8; 62.1]
75-79	78.9 ± 2.8 [73.2; 84.3]	99.4 ± 3.9 [91.8; 106.9]	68.5 ± 2.7 [63.1; 73.8]
80-84	67.3 ± 2.3 [62.8; 71.7]	89.3 ± 3.1 [83.1; 95.5]	58.5 ± 2.5 [53.6; 63.4]
85+	43.1 ± 1.9 [39.4; 46.9]	65.2 ± 5.3 [54.8; 75.6]	36.1 ± 1.9 [32.3; 39.9]
<50	1.2 ± 0.03 [1.2; 1.3]	1.2 ± 0.03 [1.2; 1.3]	1.3 ± 0.04 [1.2; 1.4]
≥ 50	37.0 ± 0.7 [35.6; 38.4]	39.6 ± 0.9 [37.8; 41.4]	35.1 ± 0.6 [33.9; 36.3]
CIR***	8.9 ± 0.2 [8.5; 9.4]	8.4 ± 0.3 [7.8; 8.9]	9.5 ± 0.2 [9.1; 9.9]
ASR****	9.4 ± 0.2 [9.1; 9.7]	10.8 ± 0.2 [10.3; 11.3]	8.6 ± 0.1 [8.3; 8.9]

*SE, Standard error; **CI, Confidence interval; ***CIR, Crude incidence rate; ****ASR, Age standardized incidence rate.

Male and female incidence patterns were broadly similar across the life course, but male rates became consistently higher from late middle age onward. The largest sex differentials were observed at 70–74 years (81.8 vs. 59.4 per 100,000), 75–79 years (99.4 vs. 68.5 per 100,000), and 80–84 years (89.3 vs. 58.5 per 100,000) for men and women, respectively. Below age 50, incidence remained low and sex differences were minimal (**Table 2**).

At the aggregated level, individuals aged ≥50 years had an incidence rate of 37.0 per 100,000, compared with 1.2 per 100,000 among those aged<50 years, corresponding to an approximately 30-fold difference. The crude incidence rate was 8.9 per 100,000, whereas the age-standardized incidence rate reached 9.4 per 100,000 overall, with higher ASR in men (10.8) than in women (8.6 per 100,000) (**Table 2**).

### Temporal trends in colon cancer incidence by sex and age group

Joinpoint regression identified a non-linear but overall increasing trend in colon cancer incidence in Kazakhstan between 2005 and 2024. In the total population, incidence increased significantly during 2005-2015 (APC =+ 2.8%), plateaued with a non-significant decline during 2015-2021, and then rose sharply in 2021-2024 (APC =+ 7.2%), resulting in a significant overall upward trend (AAPC =+ 2.3%; p<0.001) (**Table 3**).

**Table 3:** Temporal trends in colon cancer incidence in Kazakhstan by sex and age groups, 2005– 2024: Joinpoint regression analysis.

Group		Segment (years)	APC* (%)	95% CI**	*p*-value***
Both sexes	Overall	2005-2015	+2.8	[2.1; 4.2]	0.003
		2015-2021	−1.1	[−5.1; 0.4]	0.129
		2021-2024	+7.2	[3.3; 13.2]	<0.001
		AAPC****	+2.3	[1.8; 2.7]	<0.001
	<50 years	2005-2015	−1.9	[−10.1; 0.0]	0.046
		2015-2024	+2.0	[−0.1; 10.9]	0.060
		AAPC****	−0.1	[−1.1; 0.9]	0.893
	≥ 50 years	2005-2015	+2.3	[1.4; 4.0]	0.012
		2015-2021	−2.6	[−7.8; −0.8]	0.018
		2021-2024	+6.9	[1.8; 14.2]	0.010
		AAPC****	+1.5	[0.9; 2.0]	<0.001
Male	Overall	2005-2015	+3.5	[2.5; 5.3]	0.002
		2015-2021	−1.2	[−5.8; 0.5]	0.146
		2021-2024	+9.0	[4.4; 16.3]	<0.001
		AAPC****	+2.8	[2.3; 3.4]	<0.001
	<50 years	2005-2024	−0.1	[−1.5; 1.5]	0.947
		AAPC****	−0.1	[−1.5; 1.5]	0.947
	≥ 50 years	2005-2015	+2.8	[1.8; 4.5]	<0.001
		2015-2021	−2.7	[−7.1; −0.9]	0.004
		2021-2024	+8.7	[4.1; 15.7]	<0.001
		AAPC****	+1.9	[1.4; 2.5]	<0.001
Female	Overall	2005-2016	+2.2	[1.5; 4.2]	0.023
		2016-2020	−2.5	[−5.9; 0.6]	0.106
		2020-2024	+4.8	[1.9; 10.9]	0.004
		AAPC****	+1.8	[1.3; 2.3]	<0.001
	<50 years	2005-2014	−3.0	[−13.5; −0.1]	0.040
		2014-2024	+2.4	[−0.1; 13.8]	0.054
		AAPC****	−0.2	[−1.4; 1.1]	0.755
	≥ 50 years	2005-2016	+1.8	[0.6; 4.1]	0.042
		2016-2020	−4.4	[−8.7; 0.1]	0.052
		2020-2024	+4.3	[−0.1; 12.5]	0.052
		AAPC****	+1.0	[0.4; 1.7]	0.017

*APC, Annual percentage change; **CI, Confidence interval; ***p, level of significance; ****AAPC, Average Annual percent change.

This increase was driven mainly by individuals aged ≥50 years, in whom incidence showed a significant long-term rise (AAPC =+ 1.5%; p<0.001), despite an interim decline in 2015-2021. By contrast, the<50-year population showed no significant overall temporal change (AAPC=−0.1%; p=0.893), indicating the absence of a sustained early-onset increase at the population level (**Table 3**).

Sex-specific analysis demonstrated a stronger increase in men than in women. Male incidence showed the steepest long-term rise (AAPC =+ 2.8%) and the most pronounced post-2021 increase (APC =+ 9.0%), whereas female incidence increased more moderately (AAPC =+ 1.8%). In the younger age group, incidence remained stable in men and showed no significant long-term change in women, despite an early decline followed by a later upward tendency. In contrast, among individuals aged ≥50 years, both sexes demonstrated significant long-term increases, with a greater magnitude in men than in women (**Table 3**).

### Regional distribution, structural changes, and spatial characteristics of colon cancer incidence in Kazakhstan, 2005–2024

In 2024, a total of 2,199 new colon cancer cases were registered in Kazakhstan, of which 218 (9.9%) occurred in individuals aged<50 years and 1,981 (90.1%) in those aged ≥50 years.

The largest shares of incident cases were observed in Almaty city (11.8%), Karaganda region (11.2%), East Kazakhstan region (9.6%), and Kostanay region (8.8%), whereas the lowest contributions were recorded in Mangystau (2.2%), Atyrau (2.5%), and Kyzylorda (2.5%) (**Table 4A**).

**Table 4A:** Regional distribution and structural changes of colon cancer cases in Kazakhstan, 2005–2024.

№	Region	Total cases, 2024 (n, %)	Δ share, p.p.*	<50 years (n, %)	Δ<50, p.p.*	≥50 years (n, %)	Δ ≥50, p.p.*
1	Akmola	152 (6.9%)	+0.9	12 (5.5%)	+2.1	140 (7.1%)	+0.6
2	Aktobe	115 (5.2%)	+1.8	16 (7.3%)	+4.4	99 (5.0%)	+1.6
3	Almaty	155 (7.0%)	+2.4	22 (10.1%)	+5.0	133 (6.7%)	+2.2
4	Atyrau	55 (2.5%)	+0.4	7 (3.2%)	+0.3	48 (2.4%)	+0.4
5	East Kazakhstan	211 (9.6%)	−7.2	19 (8.7%)	−3.9	192 (9.7%)	−7.8
6	Zhambyl	80 (3.6%)	+0.6	8 (3.7%)	+1.4	72 (3.6%)	+0.5
7	West Kazakhstan	83 (3.8%)	+0.9	4 (1.8%)	−2.2	79 (4.0%)	−0.8
8	Karaganda	247 (11.2%)	−1.4	20 (9.2%)	−3.9	227 (11.5%)	−1.0
9	Kostanay	194 (8.8%)	−0.9	21 (9.6%)	−0.1	173 (8.7%)	−1.0
10	Kyzylorda	56 (2.5%)	+0.2	7 (3.2%)	−1.4	49 (2.5%)	+0.6
11	Mangystau	48 (2.2%)	+1.1	6 (2.8%)	+0.5	42 (2.1%)	+1.2
12	Pavlodar	135 (6.1%)	−2.4	6 (2.8%)	−8.6	129 (6.5%)	−1.5
13	North Kazakhstan	115 (5.2%)	−1.4	10 (4.6%)	−1.7	105 (5.3%)	−1.4
14	South Kazakhstan	131 (6.0%)	+2.2	15 (6.9%)	+1.2	116 (5.9%)	+2.5
15	Almaty city	259 (11.8%)	+1.4	28 (12.8%)	+3.7	231 (11.7%)	+1.1
16	Astana city	163 (7.4%)	+3.0	17 (7.8%)	+3.2	146 (7.4%)	+3.1
17	Kazakhstan	2199 (100.0%)	—	218 (100.0%)	—	1981 (100.0%)	—

*p.p., percentage points. Changes represent the difference in the proportion of total Kazakhstan cases attributable to each region between 2005 and 2024, expressed in percentage points.

Between 2005 and 2024, the regional structure of incident cases shifted toward Astana city (+3.0 percentage points), Almaty region (+2.4 p.p.), and South Kazakhstan region (+2.2 p.p.), while the relative contribution of East Kazakhstan (−7.2 p.p.), Pavlodar (−2.4 p.p.), Karaganda (−1.4 p.p.), and North Kazakhstan (−1.4 p.p.) declined. Among patients aged<50 years, the largest structural increases were observed in Almaty region (+5.0 p.p.), Aktobe region (+4.4 p.p.), Almaty city (+3.7 p.p.), and Astana city (+3.2 p.p.), whereas the sharpest declines occurred in Pavlodar (−8.6 p.p.), East Kazakhstan (−3.9 p.p.), and Karaganda (−3.9 p.p.) (**Table 4A**).

Substantial regional heterogeneity in colon cancer incidence persisted in 2024. Nationally, the crude incidence rate (CR) was 11.0 ± 0.2 per 100,000, whereas the age-standardized rate (ASR) was 10.0 ± 0.2, indicating an overall contribution of population ageing to the observed burden (**Table 4B**). The highest CR values were recorded in Kostanay (23.4 ± 1.7), North Kazakhstan (21.7 ± 2.0), and Akmola (19.3 ± 1.6), while the highest ASRs were observed in Kostanay (15.2 ± 1.1), Akmola (13.9 ± 1.2), Astana city (13.6 ± 1.1), Karaganda (13.5 ± 0.9), and North Kazakhstan (13.0 ± 1.3). The lowest rates remained concentrated in the southern regions, particularly South Kazakhstan, Almaty region, and Zhambyl (**Table 4B**).

**Table 4B:** Regional changes in colon cancer incidence rates in Kazakhstan between 2005 and 2024.

№	Region	CR* 2024 ± SE	ASR** 2024 ± SE	ΔCR (%)***	ΔASR (%)***	Gap***
1	Akmola	19.3 ± 1.6	13.9 ± 1.2	+100.1	+53.5	+5.4
2	Aktobe	12.2 ± 1.1	11.9 ± 1.1	+108.9	+80.3	+0.3
3	Almaty	7.0 ± 0.6	6.5 ± 0.5	+101.9	+63.3	+0.5
4	Atyrau	7.8 ± 1.1	9.6 ± 1.4	+46.2	+27.4	−1.8
5	East Kazakhstan	15.8 ± 1.1	10.2 ± 0.7	+13.6	−13.6	+5.6
6	Zhambyl	6.5 ± 0.7	6.5 ± 0.7	+81.1	+35.4	0.0
7	West Kazakhstan	12.0 ± 1.3	9.9 ± 1.1	+30.0	+4.2	+2.1
8	Karaganda	18.2 ± 1.2	13.5 ± 0.9	+61.8	+29.2	+4.7
9	Kostanay	23.4 ± 1.7	15.2 ± 1.1	+82.4	+32.7	+8.2
10	Kyzylorda	6.7 ± 0.9	7.3 ± 1.0	+51.5	+22.9	−0.6
11	Mangystau	6.1 ± 0.9	8.8 ± 1.3	+72.7	+53.8	−2.7
12	Pavlodar	17.9 ± 1.5	12.1 ± 1.1	+31.8	−6.6	+5.8
13	North Kazakhstan	21.7 ± 2.0	13.0 ± 1.3	+82.5	+30.4	+8.7
14	South Kazakhstan	3.9 ± 0.3	5.1 ± 0.5	+91.5	+66.6	−1.2
15	Almaty city	11.6 ± 0.7	10.5 ± 0.7	+15.2	+8.7	+1.1
16	Astana city	11.4 ± 0.9	13.6 ± 1.1	+18.3	+7.7	−2.2
17	Kazakhstan	11.0 ± 0.2	10.0 ± 0.2	+39.6	+17.4	+1.0

*CR, crude incidence rate; **ASR, age-standardized incidence rate; Gap***, absolute difference between crude and age-standardized incidence rates in 2024; positive values suggest a contribution of population ageing.

***Percentage change was calculated as the relative change between 2005 and 2024 using the formula: ((rate in 2024 − rate in 2005)/rate in 2005) × 100. Values are presented as rates ± standard error (SE). Standard errors were calculated assuming a Poisson distribution of incident cases.

From 2005 to 2024, crude incidence increased in all regions, with the largest rises in Aktobe (+108.9%), Almaty region (+101.9%), and Akmola (+100.1%). Age-standardized incidence also increased in most territories, most markedly in Aktobe (+80.3%), South Kazakhstan (+66.6%), Almaty region (+63.3%), and Mangystau (+53.8%). In contrast, East Kazakhstan (−13.6%) and Pavlodar (−6.6%) were the only regions showing declines in ASR despite rising crude rates, indicating that part of the crude increase in these territories was driven by demographic ageing rather than by increasing age-specific risk (**Table 4B**). This interpretation is supported by the largest positive crude–standardized gaps in North Kazakhstan (+8.7), Kostanay (+8.2), Pavlodar (+5.8), East Kazakhstan (+5.6), and Akmola (+5.4), whereas negative gaps in Mangystau (−2.7), Astana city (−2.2), Atyrau (−1.8), and South Kazakhstan (−1.2) reflected a relatively younger regional age structure. The difference between crude and standardized incidence rates further highlighted the uneven influence of population age structure across regions. The largest positive crude–standardized gaps in 2024 were observed in North Kazakhstan (+8.7), Kostanay (+8.2), Pavlodar (+5.8), East Kazakhstan (+5.6), and Akmola (+5.4), indicating a pronounced contribution of older population structure to the regional incidence burden. By contrast, negative gaps were observed in Mangystau (−2.7), Astana city (−2.2), Atyrau (−1.8), and South Kazakhstan (−1.2), suggesting a relatively younger age structure in these regions (**Table 4B**).

**Figure 1:**
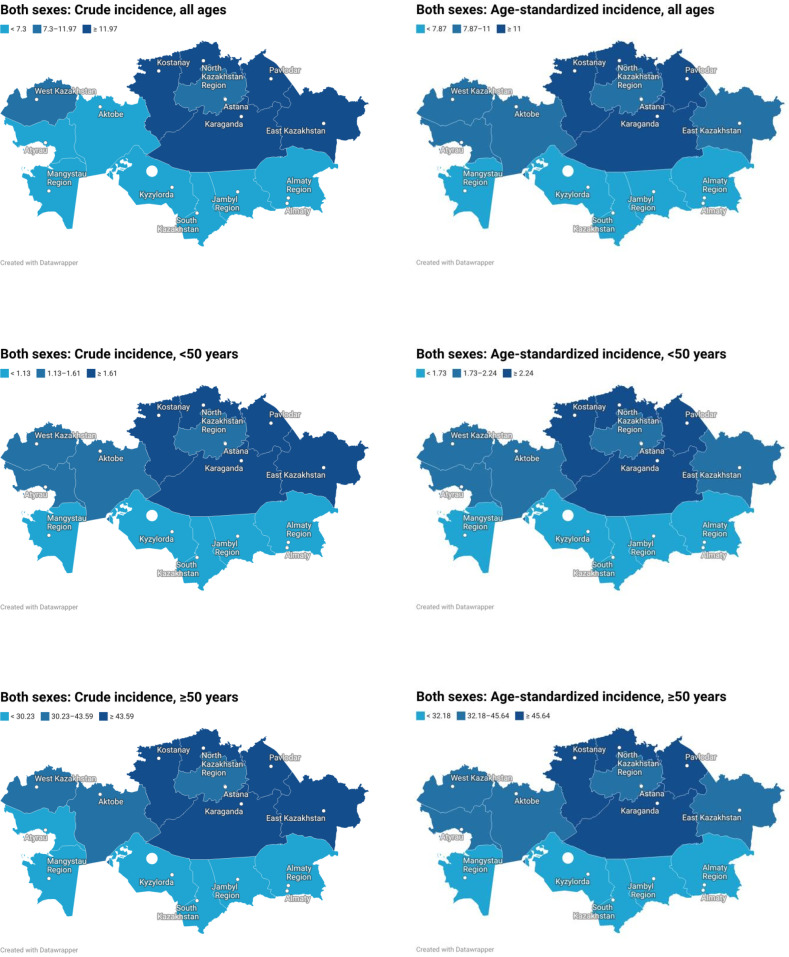
Regional distribution of crude and age-standardized colon cancer incidence rates in Kazakhstan among both sexes, overall and by age group.

Spatial analysis showed a persistent north-central high-incidence belt and a southern low-incidence belt. In the total population, ASRs ranged from 4.48 in South Kazakhstan to 14.20 in Astana city, with other high-incidence territories including Pavlodar (13.45), Almaty city (12.39), Kostanay (12.32), Karaganda (12.00), and North Kazakhstan (11.35). Age-stratified analysis showed that spatial variability was limited in individuals aged<50 years (ASR range: 1.11–2.92) but markedly amplified in those aged ≥50 years (18.06–60.80), indicating that the overall geographic pattern was driven primarily by the older population. In the<50-year group, the highest ASRs were observed in Karaganda (2.92), Kostanay (2.64), North Kazakhstan (2.55), and Pavlodar (2.40), whereas in the ≥50-year group the highest values were recorded in Astana (60.80), Pavlodar (56.45), Almaty city (52.05), Kostanay (50.70), and Karaganda (48.54) (**Figure 1**).

**Figure 2:**
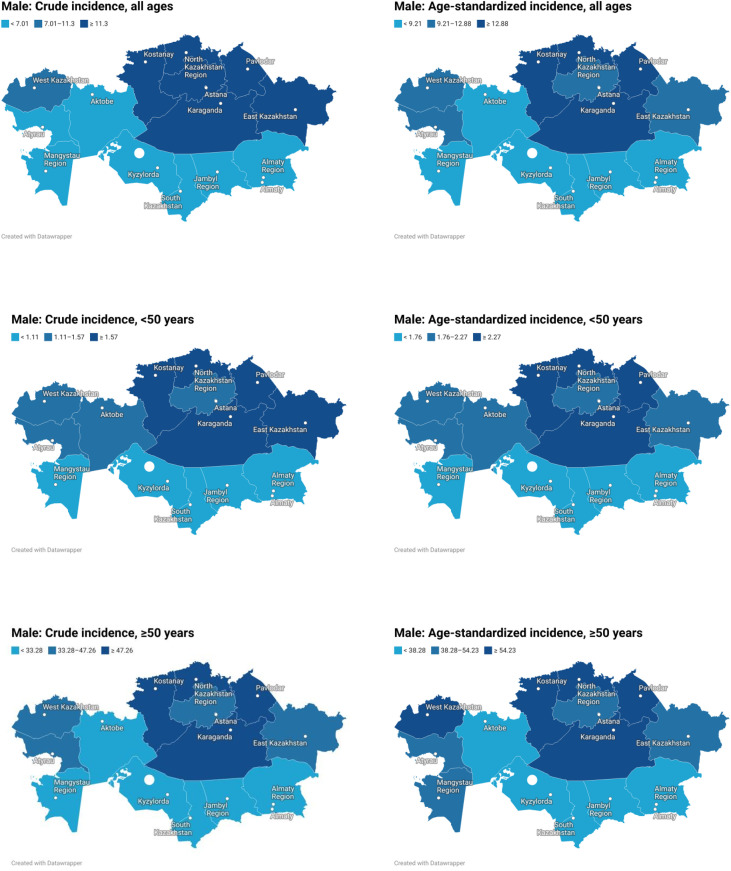
Regional distribution of crude and age-standardized colon cancer incidence rates in Kazakhstan among male population, overall and by age group.

**Figure 3:**
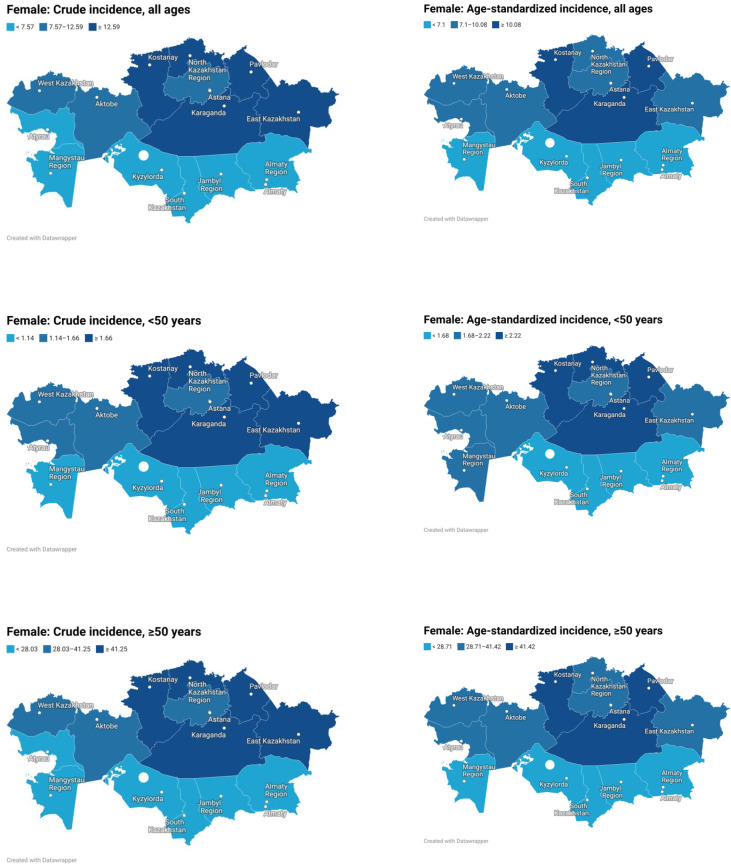
Regional distribution of crude and age-standardized colon cancer incidence rates in Kazakhstan among female population, overall and by age group.

Sex-specific analysis showed that the mean crude incidence rate was slightly higher in women than in men (10.08 vs. 9.16 per 100,000), whereas the mean ASR was higher in men than in women (11.05 vs. 8.59), indicating that the apparent crude excess in women was largely attributable to their older age structure. Before age 50, sex differences were minimal (ASR 2.01 in men vs. 1.95 in women), but after age 50 they became pronounced (46.26 vs. 35.07). Among men, the highest ASRs were observed in Pavlodar (16.03), Astana (15.61), Almaty city (14.70), North Kazakhstan (13.94), and Kostanay (13.92), whereas among women the highest values were recorded in Astana (13.61), Pavlodar (12.23), Kostanay (11.46), Karaganda (11.32), and Almaty city (11.14) (**Figures 2**, **3**).

### Hotspot and coldspot regions

**Figure 4:**
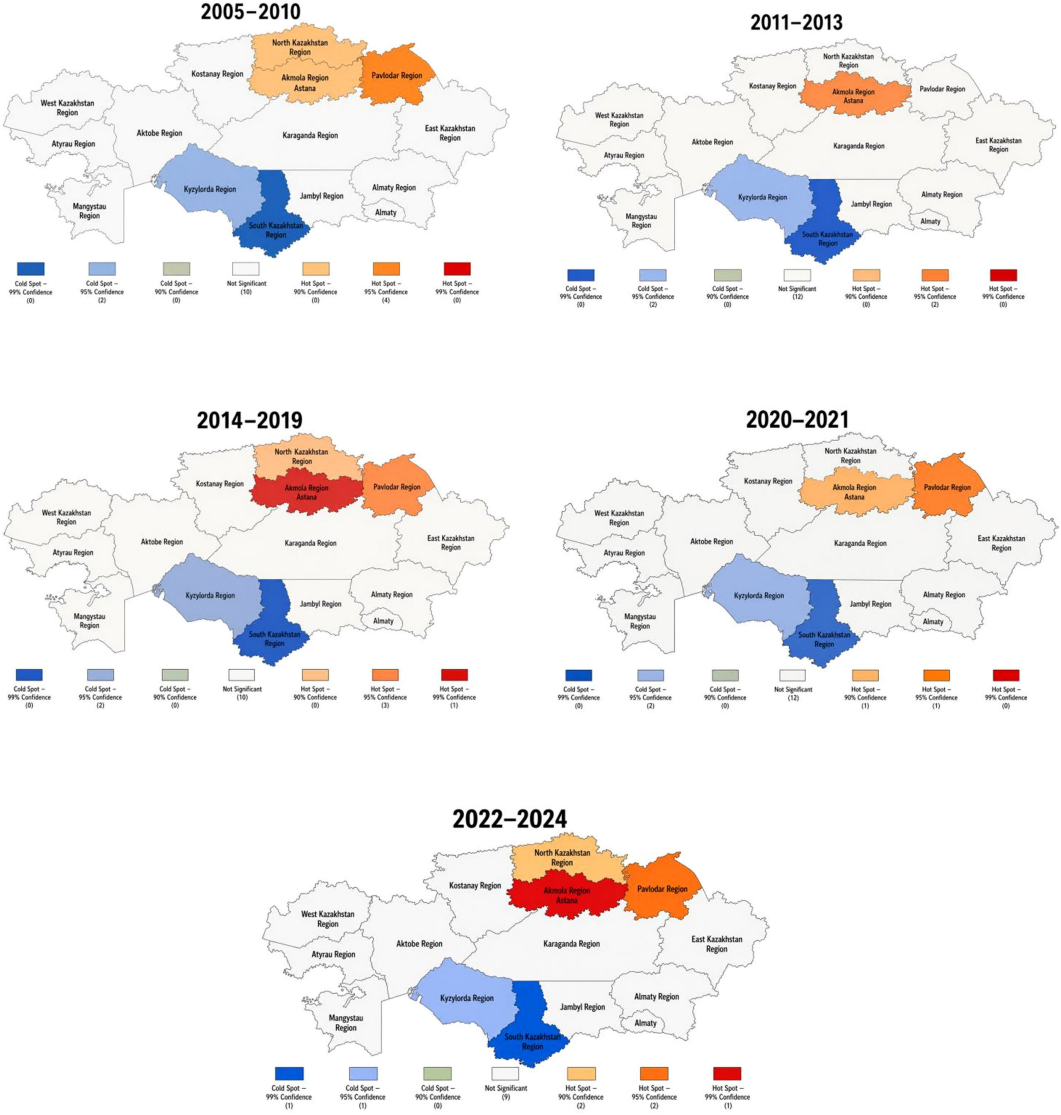
Hotspot and coldspot of average age-standardized incidence rate of colon cancer in Kazakhstan.

The Getis–Ord Gi* analysis identified statistically significant spatial clustering patterns. Northern and central regions demonstrated hotspot characteristics, while southern regions were identified as coldspots. These findings were consistent with the descriptive spatial distribution and support the presence of a north-south gradient. However, given the limited number of spatial units, these results should be interpreted as exploratory (**Figure 4**).

In 2005-2010, significant hotspots were identified in North Kazakhstan, Akmola, Pavlodar, and Astana, whereas South Kazakhstan and Kyzylorda formed significant coldspots. In 2011-2013, hotspot clustering became more localized and was confined to Akmola and Astana, while the southern coldspots remained unchanged. During 2014-2019, hotspot clustering intensified again, with Akmola showing the strongest concentration, and North Kazakhstan, Astana, and Pavlodar remaining significant high-incidence areas; South Kazakhstan and Kyzylorda continued to represent stable coldspots. In 2020-2021, the hotspot pattern narrowed to Akmola and Astana, whereas the southern coldspots persisted. In 2022-2024, the north-central hotspot belt re-emerged more clearly: Akmola showed the strongest clustering, Astana and North Kazakhstan remained significant hotspots, and Pavlodar and Kostanay also entered the hotspot zone, while South Kazakhstan and Kyzylorda remained persistent coldspots. Overall, the spatial clustering analysis confirmed a stable north–south gradient, with recurrent high-incidence clusters in northern and central Kazakhstan and sustained low-incidence clusters in the south.

### Regional temporal trends in colon cancer incidence

Joinpoint regression demonstrated marked regional heterogeneity in long-term colon cancer incidence trends across Kazakhstan. Based on crude rates, a significant increase was observed in nearly all regions over 2005–2024, with the strongest average annual growth in Aktobe (AAPC =+ 3.9%; p<0.001), Atyrau (+3.6%; p=0.001), Akmola and Zhambyl (both +3.3%). In contrast, the weakest crude-rate increases were recorded in West Kazakhstan (AAPC =+ 0.3%; p=0.588) and East Kazakhstan (+1.0%; p=0.032) (**Table 4C**).

**Table 4C:** Temporal trends in colon cancer incidence in Kazakhstan by regions, 2005-2024: Joinpoint regression analysis.

№	Region	Crude rate			Age-standardized rate		
		AAPC (%)*	95% CI**	p***	AAPC (%)*	95% CI**	p***
1	Akmola	+3.3	[2.0;4.8]	<0.001	+1.9	[0.6;3.4]	0.005
2	Aktobe	+3.9	[2.1;5.1]	<0.001	+1.8	[0.3;3.6]	0.023
3	Almaty	+3.1	[1.3;5.3]	0.001	+1.9	[0.1;4.2]	0.040
4	Atyrau	+3.6	[1.5;6.4]	0.001	+2.6	[0.8;4.9]	0.006
5	East Kazakhstan	+1.0	[0.1;2.0]	0.032	−1.0	[−1.5;−0.3]	0.007
6	Zhambyl	+3.3	[1.1;3.9]	<0.001	+2.1	[0.1;4.4]	0.046
7	West Kazakhstan	+0.3	[−1.0;1.7]	0.588	−0.8	[−2.0;0.4]	0.172
8	Karaganda	+2.1	[1.1;3.3]	<0.001	+0.9	[−0.2;2.1]	0.105
9	Kostanay	+3.2	[2.0;4.7]	<0.001	+1.9	[0.7;2.9]	<0.001
10	Kyzylorda	+2.8	[1.1;4.9]	0.002	+1.0	[−0.8;3.3]	0.232
11	Mangystau	+3.3	[1.5;5.9]	<0.001	+1.6	[0.0;4.0]	0.052
12	Pavlodar	+1.5	[0.6;2.5]	0.001	−0.2	[−1.2;0.9]	0.767
13	North Kazakhstan	+2.1	[1.0;3.3]	<0.001	+1.9	[0.5;3.4]	0.008
14	South Kazakhstan	+3.2	[1.9;4.8]	<0.001	+2.5	[0.8;4.9]	0.005
15	Almaty city	+1.2	[0.2;2.3]	0.032	+1.0	[0.0;2.4]	0.047
16	Astana city	+1.3	[0.1;2.7]	0.030	+0.5	[−1.6;4.3]	0.381

*AAPC, Average annual percentage change; **95% CI, confidence interval; ***p-value for AAPC (Joinpoint regression).

After age standardization, the pattern became more differentiated. Significant long-term increases in ASR were retained in Atyrau (AAPC =+ 2.6%; p=0.006), South Kazakhstan (+2.5%; p=0.005), Zhambyl (+2.1%; p=0.046), and Akmola, Almaty, Kostanay, and North Kazakhstan (all approximately +1.9% annually). By contrast, age-standardized incidence declined significantly in East Kazakhstan (AAPC=−1.0%; p=0.007), whereas Pavlodar (−0.2%; p=0.767) and West Kazakhstan (−0.8%; p=0.172) showed non-significant downward tendencies. Non-significant ASR trends were also observed in Karaganda, Kyzylorda, Mangystau, and Astana city, despite positive crude-rate changes in these territories (**Table 4C**).

The contrast between crude and age-standardized trends was especially informative in the northern and eastern regions. In East Kazakhstan, crude incidence increased slightly while ASR declined significantly, and in Pavlodar and West Kazakhstan, positive or near-stable crude trends corresponded to flat or negative standardized trends. This divergence indicates that in these regions, the observed long-term increase in crude incidence was largely influenced by demographic ageing rather than by a sustained increase in age-specific risk. In contrast, regions such as Atyrau, South Kazakhstan, Akmola, and Kostanay demonstrated significant positive trends in both crude and standardized rates, suggesting a genuine increase in incidence beyond the effect of population age structure (**Table 4C**).

Among the two major cities, Almaty city showed a modest but significant increase in both crude and standardized incidence (AAPC =+ 1.2% and +1.0%, respectively), whereas in Astana city the crude trend increased significantly (AAPC =+ 1.3%; p=0.030) but the standardized trend did not reach statistical significance (AAPC =+ 0.5%; p=0.381). Overall, these findings indicate that regional growth in colon cancer incidence across Kazakhstan was not uniform and reflected different underlying mechanisms, including both true increases in age-specific risk and the varying contribution of population ageing (**Table 4C**).

To further disentangle the underlying temporal dynamics, age–period–cohort analysis was performed.

### Age-period-cohort analysis of colon cancer incidence in Kazakhstan

**Figure 5:**
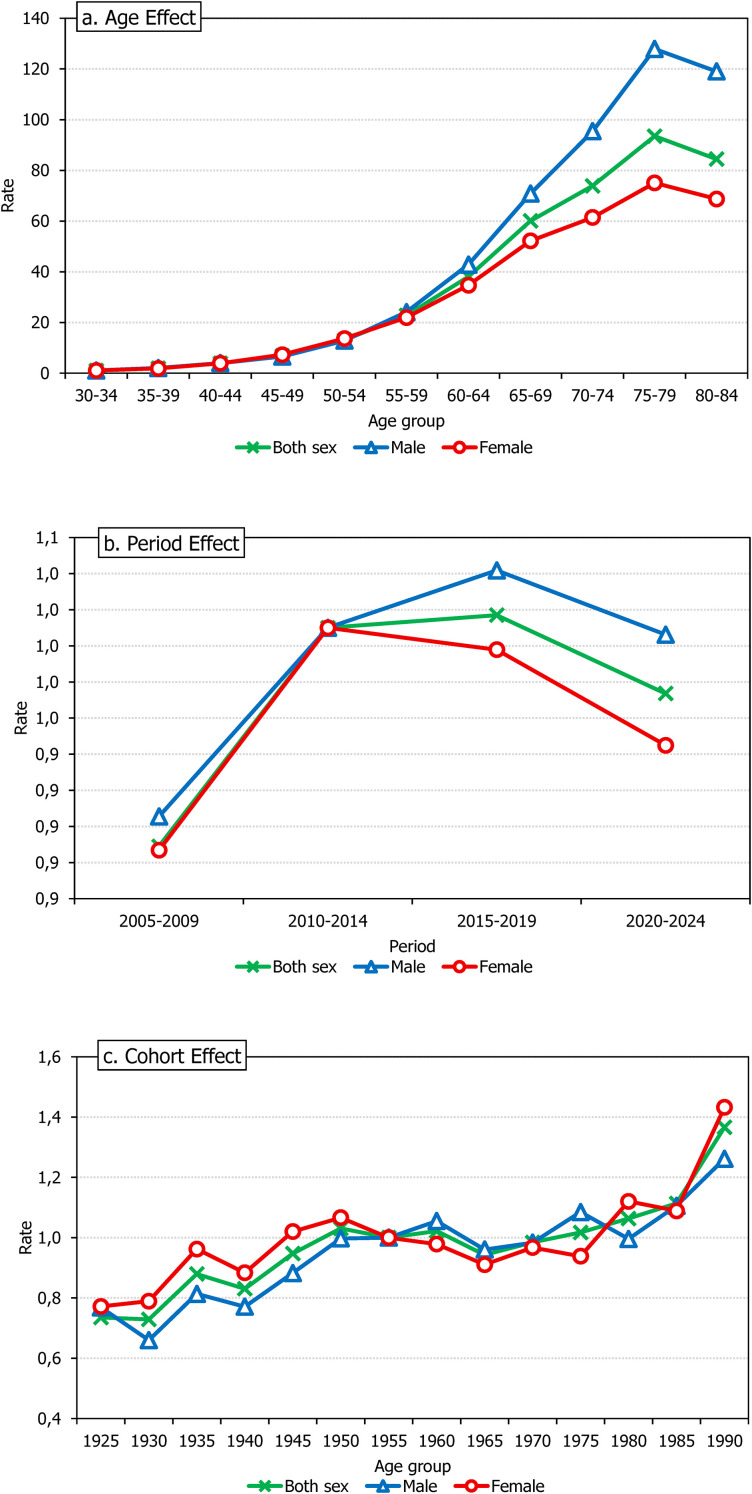
Age–period–cohort patterns of colon cancer incidence in Kazakhstan, 2005–2024: overall and sex-specific models. **(a)** Age Effect; **(b)** Period Effect; **(c)** Cohort Effect.

The age-period-cohort analysis showed a strong age effect across all models, with incidence increasing steeply with advancing age. In the combined-sex model, the net drift was +0.57% per year (95% CI: 0.23-0.91; p=0.001), and local drifts differed significantly across age groups (p<0.001), with the strongest increases observed at ages 65–84 years. The longitudinal age curve rose from 1.07 per 100,000 at ages 30–34 years to 93.51 at ages 75–79 years, followed by a slight decline thereafter (**Figure 5A**).

Among women, the net drift was +0.36% per year (95% CI: −0.06 to 0.79; p=0.096), indicating no significant overall linear increase, although age-specific temporal variation remained significant (p<0.001). Positive local drifts were concentrated in older age groups, while a significant decline was observed at ages 55–59 years (−0.99% per year). The longitudinal age curve increased from 1.08 to 75.05 per 100,000.

Among men, the net drift was +0.70% per year (95% CI: 0.28-1.13; p=0.001), indicating a significant upward trend. The strongest increases were concentrated at ages 60–79 years, and the longitudinal age curve rose from 1.1 per 100,000 at ages 30–34 years to 127.9 at ages 75–79 years.

Period effects were significant in all models. Compared with 2010-2014, incidence was significantly lower in 2005–2009 in the combined-sex, male, and female models; among women, a further reduction was also observed in 2020-2024 (**Figure 5B**). Cohort effects were likewise significant, with earlier birth cohorts showing lower risks than the 1955 reference cohort, whereas most later cohorts did not differ significantly (**Figure 5C**). Overall, the Age-period-cohort analysis indicates that the increase in colon cancer incidence in Kazakhstan was driven primarily by older age groups, particularly among men.

## Discussion

This nationwide study provides an integrated description of colon cancer incidence in Kazakhstan over a 20-year period by combining descriptive epidemiology, spatial assessment, Joinpoint trend analysis, hotspot detection, and age-period-cohort modelling. Several findings stand out. First, colon cancer incidence showed a persistent increase over time, although the rise was not linear and was interrupted by a temporary decline during the COVID-19 period. Globally, the burden of colorectal cancer has risen sharply over the past three decades. Data from Global Burden of Diseases 2019 showed that global age-standardized incidence of early-onset colorectal cancer increased from 3.05 (3.03, 3.07) to 3.85 (3.83, 3.86) per 100,000 during 1990 and 2019., with the incidence was higher in countries with high socioeconomic levels, and increased drastically in countries in East Asia and Caribbean (17). Kazakhstan fits within this middle-SDI cluster, reflecting rising incidence trends driven by aging populations, westernized lifestyles, and limited early detection infrastructure. Moreover, Kazakhstan’s recent national registry (2014–2023) shows colorectal cancer ASR peaking around 2016 (23.19 per 100,000) and 2017 (22.88 per 100,000), dipping during the pandemic, and then rebounding-paralleling our periodization findings of colon cancer ASR, particularly the COVID-19–related dip in 2020-2021 (16).

Second, the disease burden was strongly age-dependent: the overwhelming majority of incident cases occurred in individuals aged 50 years and older, and both the spatial gradient and temporal increase were driven mainly by this older population.

Third, a stable north–south contrast was observed, with recurrent high-incidence areas in the northern and central regions and persistent low-incidence areas in the south.

Finally, incidence was higher in men after age standardization, and the sex difference became pronounced only after age 50, indicating that the male excess was largely a feature of later-onset disease rather than early-onset incidence. This aligns with broader national findings: a retrospective study (2009-2018) reported rising colorectal cancer incidence in Kazakhstan – from 14.8 to 17.7 per 100,000 – driven by demographic shifts and increased disease risk (15). The observed regional disparities likely reflect variations in healthcare access, screening uptake, and lifestyle factors.

The age-specific findings are central to the interpretation of the national trend. In the updated descriptive analysis, 90.1% of newly diagnosed cases in 2024 occurred in individuals aged ≥50 years, and age-period-cohort analysis modelling showed that the strongest positive local drifts were concentrated in ages 65–84 years, with a significant overall net drift in the combined-sex and male models but not in women. Together, these results indicate that the long-term increase in colon cancer incidence in Kazakhstan was driven predominantly by population ageing and by rising incidence concentrated in older adults rather than by a generalized shift across all ages. This interpretation is further supported by the weak spatial variability and low absolute rates in the<50-year group, in contrast to the marked regional divergence observed in older adults. Thus, although early-onset colon cancer remains epidemiologically important, it currently contributes relatively little to the national burden in Kazakhstan compared with late-onset disease.

Kazakhstan’s national data from 2014 to 2023 further confirms these upward trends: overall colorectal cancer ASR increased from approximately 18.9 to 20.8 per 100,000, with higher rates among males (from 22.7 to 25.3) than females (from 16.6 to 18.0) (16). In our study crude rates were slightly higher in women, but age-standardized rates were clearly higher in men, indicating that the apparent female excess in crude incidence was largely explained by older age structure rather than by higher underlying risk. Before age 50, male and female incidence was very similar, whereas after age 50 a clear male excess emerged. This male predominance in colon cancer ASR (11.8 per 100,000 in male and 9 per 100,000 in female) is consistent with global trends, where men often exhibit higher colorectal cancer incidence. In 2020, colorectal cancer ASR was 19.5 per 100,000 (23.4 in males, 16.2 in females) (3, 4).

A major contribution of this study is the demonstration of persistent spatial heterogeneity. Both descriptive maps and Gi* statistics showed a stable north-central high-incidence belt and a southern low-incidence belt throughout the study period. The recurrence of hotspots in Akmola, Astana, North Kazakhstan, Pavlodar, and later Kostanay suggests that the observed clustering was not random or transient. At the same time, the persistence of coldspots in Kyzylorda and South Kazakhstan indicates that low-incidence regions also remained spatially stable across screening-related and pandemic-related phases. Importantly, the contrast between crude and age-standardized measures showed that demographic structure played a substantial role in some territories. Regions such as North Kazakhstan, Kostanay, Pavlodar, and East Kazakhstan had large positive crude–standardized gaps, implying an important contribution of older population structure to the crude burden.

National incidence rose after the introduction of organized colorectal cancer screening, remained elevated during the program expansion phase, declined during the COVID-19 disruption, and rebounded thereafter. This sequence is compatible with changes in case ascertainment and diagnostic access. In particular, the early post-implementation increase may reflect enhanced detection of previously undiagnosed disease, whereas the 2020–2021 decline is consistent with reduced screening coverage, delayed colonoscopy, and lower diagnostic throughput during the pandemic. However, these temporal alignments should not be interpreted as direct proof of screening effectiveness.

These results also help refine the interpretation of regional trend differences. In most regions, crude incidence increased more strongly than age-standardized incidence, indicating that demographic ageing explained part of the rise. In East Kazakhstan and Pavlodar, age-standardized incidence even declined despite increasing crude rates, reinforcing the importance of separating demographic from epidemiologic drivers. By contrast, regions such as Aktobe, South Kazakhstan, Almaty region, and Mangystau showed marked increases even after age standardization, suggesting that factors beyond ageing – including changing exposure patterns, diagnostic expansion, or improved registration – may have contributed. These regional differences indicate that Kazakhstan is not experiencing a single uniform national process but rather several overlapping regional trajectories.

This study has limitations that should be explicitly acknowledged. First, its ecological design does not allow causal inference at the individual level. Second, the dataset lacked information on stage at diagnosis, treatment, colonoscopy use, screening participation, pathology, survival, and mortality specific to colon cancer; therefore, interpretations regarding screening impact or improved early detection must remain cautious. Third, the registry-based design did not include individual lifestyle or socioeconomic factors, so biological and social explanations for regional disparities remain inferential. Fourth, the hotspot analysis was conducted at the regional rather than district level, which limits spatial resolution and means that Gi* results should be interpreted as exploratory macro-level clustering rather than fine-scale local concentration. Finally, the analysis was restricted to colon cancer and cannot be generalized directly to rectal cancer or total colorectal cancer burden.

Despite these limitations, the study has several important strengths. It is based on nationwide population-level data over two decades, uses both crude and age-standardized rates, explicitly separates early-onset and later-onset disease, incorporates sex-specific and region-specific analyses, and combines descriptive cartography with formal hotspot detection, Joinpoint modelling, and age-period-cohort analysis. This integrated framework provides a more complete picture of colon cancer incidence in Kazakhstan than simple national trend descriptions alone.

In conclusion, the contemporary epidemiology of colon cancer incidence in Kazakhstan appears to be shaped by three dominant forces: population ageing, persistent north–south spatial inequality, and sex-specific excess concentrated in older men. Screening-related and pandemic-related phases likely influenced case detection, but the present data do not justify strong causal claims regarding program effectiveness. The practical implication is that colon cancer control in Kazakhstan should prioritize older adults, especially men, while also addressing regional inequities in diagnostic capacity and access to care. Future studies should link incidence data with stage, mortality, screening participation, and treatment indicators to determine whether the observed increase primarily reflects improved detection, changing risk exposure, or both.

## Data Availability

The original contributions presented in the study are included in the article/supplementary material. Further inquiries can be directed to the corresponding author.
